# Ethane-1,2-diaminium dipicrate dihydrate

**DOI:** 10.1107/S1600536811004855

**Published:** 2011-02-16

**Authors:** Jerry P. Jasinski, Ray J. Butcher, Q. N. M. Hakim Al-arique, H. S. Yathirajan, B. Narayana

**Affiliations:** a Department of Chemistry, Keene State College, 229 Main Street, Keene, NH 03435-2001, USA; b Department of Chemistry, Howard University, 525 College Street NW, Washington, DC 20059, USA; c Department of Studies in Chemistry, University of Mysore, Manasagangotri, Mysore 570 006, India; d Department of Studies in Chemistry, Mangalore University, Mangalagangotri 574 199, India

## Abstract

The title compound, C_2_H_10_N_2_^2+^·2C_6_H_2_N_3_O_7_^−^·2H_2_O, crystallizes with a complete picrate anion and half an ethyl­enediammonium dication on a mirror plane, and two half-water mol­ecules (both on a mirror plane) in the asymmetric unit. The N atoms from separate half ethyl­enediammonium dications are in near proximity to a phenolate O atom and two *o*-NO_2_ groups from the picrate anion, which, along with the water mol­ecule form N—H⋯O, O—H⋯O and weak intermolecular C—H⋯O hydrogen bonds that create cyclic patterns with graph-set descriptors *R*_2_^4^(8), *R*_4_^4^(12), and *R*_4_^4^(16). The crystal packing is strongly influenced by these inter­molecular inter­actions between symmetry-related water mol­ecules, the half ethyl­enediammonium dication and the picrate anion, forming a three-dimensional supermolecular structure.

## Related literature

For related structures, see: Muthamizhchelvan *et al.* (2005*a* ▶,*b* ▶,*c* ▶); Subashini *et al.* (2006 ▶); Narayana *et al.* (2008 ▶). For standard bond lengths, see: Allen *et al.* (1987 ▶). For picrates of biologically important molecules, see: Harrison *et al.* (2007 ▶); Swamy *et al.* (2007 ▶).
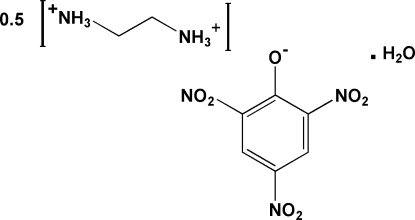


## Experimental

### 

#### Crystal data

C_2_H_10_N_2_^2+^·2C_6_H_2_N_3_O_7_^−^·2H_2_O
*M* *_r_* = 554.36
Orthorhombic, 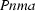

*a* = 13.4795 (4) Å
*b* = 20.4372 (7) Å
*c* = 8.0410 (3) Å
*V* = 2215.16 (13) Å^3^
*Z* = 4
Mo *K*α radiation
μ = 0.15 mm^−1^
*T* = 295 K
0.52 × 0.42 × 0.27 mm

#### Data collection

Oxford Diffraction Gemini R diffractometer
Absorption correction: multi-scan (*CrysAlis RED*; Oxford Diffraction, 2007 ▶) *T*_min_ = 0.837, *T*_max_ = 0.960
16775 measured reflections
3846 independent reflections
2322 reflections with *I* > 2σ(*I*)
*R*_int_ = 0.026

#### Refinement

*R*[*F* ^2^ > 2σ(*F* ^2^)] = 0.063
*wR*(*F* ^2^) = 0.196
*S* = 1.03
3846 reflections
207 parameters
10 restraints
H atoms treated by a mixture of independent and constrained refinement
Δρ_max_ = 0.37 e Å^−3^
Δρ_min_ = −0.27 e Å^−3^

### 

Data collection: *CrysAlis PRO* (Oxford Diffraction, 2007 ▶); cell refinement: *CrysAlis PRO*; data reduction: *CrysAlis PRO*; program(s) used to solve structure: *SHELXS97* (Sheldrick, 2008 ▶); program(s) used to refine structure: *SHELXL97* (Sheldrick, 2008 ▶); molecular graphics: *SHELXTL* (Sheldrick, 2008 ▶) and *Mercury* (Macrae *et al.*, 2006 ▶); software used to prepare material for publication: *SHELXTL*, *enCIFer* (Allen *et al.*, 2004 ▶) and *PLATON* (Spek, 2009 ▶).

## Supplementary Material

Crystal structure: contains datablocks global, I. DOI: 10.1107/S1600536811004855/om2399sup1.cif

Structure factors: contains datablocks I. DOI: 10.1107/S1600536811004855/om2399Isup2.hkl

Additional supplementary materials:  crystallographic information; 3D view; checkCIF report

## Figures and Tables

**Table 1:** Hydrogen-bond geometry (Å, °)

*D*—H⋯*A*	*D*—H	H⋯*A*	*D*⋯*A*	*D*—H⋯*A*
N4—H41⋯O2*W*^i^	0.87 (1)	2.06 (2)	2.872 (4)	156 (3)
N4—H42⋯O1	0.86 (1)	1.98 (1)	2.815 (2)	164 (2)
N4—H42⋯O7	0.86 (1)	2.41 (2)	2.9166 (16)	118 (2)
N5—H51⋯O1*W*^ii^	0.87 (1)	1.91 (1)	2.778 (3)	176 (3)
N5—H52⋯O1^iii^	0.86 (1)	2.18 (2)	2.892 (2)	140 (2)
N5—H52⋯O2^iii^	0.86 (1)	2.34 (2)	3.018 (3)	136 (2)
C3—H3⋯O6^iv^	0.93	2.53	3.337 (3)	145
C7—H7⋯O2^v^	0.96	2.39	3.128 (3)	134
C7—H7⋯O7^vi^	0.96	2.56	3.1203 (19)	117
O1*W*—H1*W*2⋯O3	0.84 (1)	2.21 (3)	3.008 (2)	159 (6)
O1*W*—H1*W*2⋯O2	0.84 (1)	2.52 (4)	3.243 (3)	145 (5)
O2*W*—H2*W*2⋯O5	0.85 (1)	2.26 (2)	3.082 (2)	163 (7)

Symmetry codes: (i) 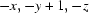; (ii) 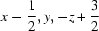; (iii) 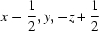; (iv) 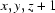; (v) 
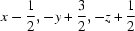
; (vi) 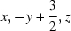.

**Fig. 1.**
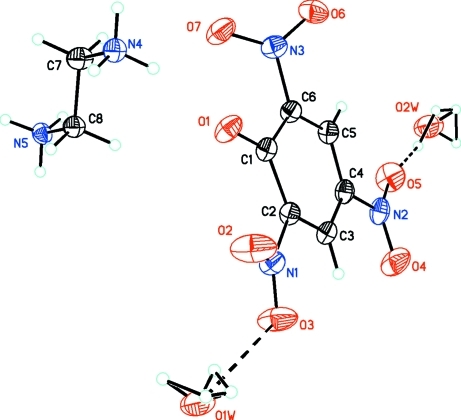
Molecular structure of the title compound, 0.5(C2H10N22+), C6H2N3O7-, H2O, showing the atom labeling scheme and 30% probability displacement ellipsoids. The asymmetric unit consists of a complete picrate anion, a half-ethylenediammonium group on a mirror plane thus producing a 0.5 di-cation (i.e. protonated at both ends), and two half-water molecules (both on a mirror plane). Dashed lines indicate O—H···O hydrogen bond interactions with a disordered water molecule..

**Fig. 2.**
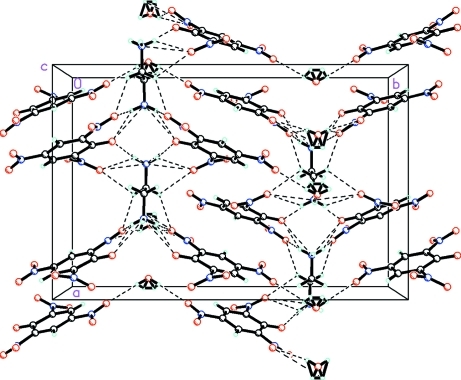
Packing diagram of the title compound viewed down the c axis. Dashed lines indicate intermolecular N—H···O and O—H···O hydrogen bonds and weak C—H···O intermolecular interactions which produces a 3-D superstructure. Disordered water molecules are displayed.

## supplementary crystallographic information

### Comment

The crystal structures of compounds similar to ethylenediammonium picrate, 3-(dimethylammonio)propanaminium dipicrate and triethylaminium picrate (Muthamizhchelvan *et al.*, 2005*a*, 2005*b*, 2005*c*), 2-amino-4,6-dimethylpyrimidinium picrate (Subashini *et al.*, 2006) and 2-aminopyrimidinium picrate (Narayana *et al.*, 2008) have been reported. In continuation of our work on picrates of biologically important molecules (Harrison *et al.*, 2007; Swamy *et al.*, 2007), we have prepared a new picrate of ethylenediammonium hydrate, [C_7_H_8_N_4_O_8_] and its crystal structure is reported.

The title compound, 0.5(C_2_H_10_N_2_^2+^), C_6_H_2_N_3_O_7_^-^, H_2_O, crystallizes with a complete picrate anion and a half-ethylenediammonium group on a mirror plane thus producing a 0.5 di-cation (*i.e.* protonated at both ends), and two half-water molecules (both on a mirror plane) in the asymmetric unit (Fig. 1). Bond distances and angles are in normal ranges (Allen *et al.*, 1998). In the picrate anion the depronated phenolate oxygen atom is slightly deviated from the plane of the benzene ring (torsion angle O1/C1/C2/C3 = 177.84 (17) Å). The twist angles between the mean plane of the benzene ring and the two *o*-NO_2_ groups are 20.3 (0)° (N1) and 39.6 (7)° (N3). The *p*-NO_2_group is twisted by 3.3 (4)° and most likely influenced by a weak hydrogen bond interaction (O2W—H2W2···O5). The deviation of the *p*-NO_2_ groups from the plane of the benzene ring is due to a network of hydrogen bond interactions with the half-ethylenediammonium di-cation involving both nitrogen atoms (N4 and N5 lying across a morror plane). The position of N4 and N5 from separate half-ethylenediammonium di-cations, in near proximity to a phenolate oxygen atom and two *o*-NO_2_ groups from the picrate anion, along with the water molecule form N—H···O, O—H···O hydrogen bonds and weak C—H···O intermolecular interactions (Table 1) that create cyclic patterns with graph-set descriptors *R*_2_^4^(8), *R*_4_^4^(12) and *R*_4_^4^(16). These intermolecular interactions of symmetry-related molecules link the cations and anions through a half-water molecule (on a mirror plane), the half-ethylenediammonium di-cation and the picrate anion forming a 3-D supermolecular structure (Fig. 2).

### Experimental

Ethylenediamine dihydrochloride (1.33 g, 0.01 mol) was dissolved in 25 ml of water. Picric acid (2.29 g, 0.01 mol) was dissolved in 50 ml of water. Both the solutions were mixed and stirred for few minutes. The formed complex was filtered and dried. Good quality crystals were grown from ethanol solution by slow evaporation (m. p.: 476–478 K). Composition: Found (Calculated): C: 30.44 (30.40); H: 2.92 (2.96); N: 20.29% (20.36%).

### Refinement

The H atoms on the water O atoms and N atoms were located by difference Fourier maps, fixed at 0.84Å (O-H) and 1.36° (O^···^O), or 0.86Å (N-H) and refined isotropically. All of the remaining H atoms were placed in their calculated positions and then refined using the riding model with Atom—H lengths of 0.93 or 0.95Å (CH). Isotropic displacement parameters for these atoms were set to 1.19–1.20 (CH) times *U*_eq_ of the parent atom.

### Crystal data

**Table:**

C_2_H_10_N_2_^2+^·2C_6_H_2_N_3_O_7_^−^·2H_2_O	*F*(000) = 1144
*M**_r_* = 554.36	*D*_x_ = 1.662 Mg m^−^^3^
Orthorhombic, *P**n**m**a*	Mo *K*α radiation, λ = 0.71073 Å
Hall symbol: -P 2ac 2n	Cell parameters from 5241 reflections
*a* = 13.4795 (4) Å	θ = 4.6–32.5°
*b* = 20.4372 (7) Å	µ = 0.15 mm^−^^1^
*c* = 8.0410 (3) Å	*T* = 295 K
*V* = 2215.16 (13) Å^3^	Chunk, pale orange
*Z* = 4	0.52 × 0.42 × 0.27 mm

### Data collection

**Table:**

Oxford Diffraction Gemini R diffractometer	3846 independent reflections
Radiation source: fine-focus sealed tube	2322 reflections with *I* > 2σ(*I*)
graphite	*R*_int_ = 0.026
Detector resolution: 10.50 pixels mm^-1^	θ_max_ = 32.5°, θ_min_ = 5.0°
φ and ω scans	*h* = −19→14
Absorption correction: multi-scan (*CrysAlis RED*; Oxford Diffraction, 2007)	*k* = −29→23
*T*_min_ = 0.837, *T*_max_ = 0.960	*l* = −11→9
16775 measured reflections	

### Refinement

**Table:**

Refinement on *F*^2^	Primary atom site location: structure-invariant direct methods
Least-squares matrix: full	Secondary atom site location: difference Fourier map
*R*[*F*^2^ > 2σ(*F*^2^)] = 0.063	Hydrogen site location: inferred from neighbouring sites
*wR*(*F*^2^) = 0.196	H atoms treated by a mixture of independent and constrained refinement
*S* = 1.03	*w* = 1/[σ^2^(*F*_o_^2^) + (0.110*P*)^2^ + 0.1282*P*] where *P* = (*F*_o_^2^ + 2*F*_c_^2^)/3
3846 reflections	(Δ/σ)_max_ < 0.001
207 parameters	Δρ_max_ = 0.37 e Å^−^^3^
10 restraints	Δρ_min_ = −0.27 e Å^−^^3^

### Special details

**Table:**

Geometry. All e.s.d.'s (except the e.s.d. in the dihedral angle between two l.s. planes) are estimated using the full covariance matrix. The cell e.s.d.'s are taken into account individually in the estimation of e.s.d.'s in distances, angles and torsion angles; correlations between e.s.d.'s in cell parameters are only used when they are defined by crystal symmetry. An approximate (isotropic) treatment of cell e.s.d.'s is used for estimating e.s.d.'s involving l.s. planes.
Refinement. Refinement of *F*^2^ against ALL reflections. The weighted *R*-factor *wR* and goodness of fit *S* are based on *F*^2^, conventional *R*-factors *R* are based on *F*, with *F* set to zero for negative *F*^2^. The threshold expression of *F*^2^ > σ(*F*^2^) is used only for calculating *R*-factors(gt) *etc*. and is not relevant to the choice of reflections for refinement. *R*-factors based on *F*^2^ are statistically about twice as large as those based on *F*, and *R*- factors based on ALL data will be even larger.

### Fractional atomic coordinates and isotropic or equivalent isotropic displacement parameters (Å^2^)

**Table:**

	*x*	*y*	*z*	*U*_iso_*/*U*_eq_	Occ. (<1)
O1	0.17845 (10)	0.65746 (6)	0.24678 (18)	0.0483 (4)	
O2	0.27913 (16)	0.67547 (9)	0.5303 (2)	0.0816 (6)	
O3	0.22821 (17)	0.62252 (9)	0.7372 (2)	0.0797 (6)	
O4	0.11929 (14)	0.40318 (8)	0.6662 (2)	0.0745 (5)	
O5	0.05313 (14)	0.37346 (7)	0.4353 (3)	0.0707 (5)	
O6	0.10573 (12)	0.51165 (8)	−0.0500 (2)	0.0590 (4)	
O7	0.05455 (14)	0.60949 (7)	0.0004 (2)	0.0663 (5)	
N1	0.23259 (12)	0.63071 (7)	0.5874 (2)	0.0435 (4)	
N2	0.09604 (12)	0.41363 (7)	0.5211 (3)	0.0472 (4)	
N3	0.09158 (12)	0.55764 (8)	0.0443 (2)	0.0452 (4)	
C1	0.16145 (11)	0.60249 (8)	0.3089 (2)	0.0350 (4)	
C2	0.18375 (12)	0.58407 (8)	0.4784 (2)	0.0343 (4)	
C3	0.16198 (12)	0.52426 (8)	0.5472 (2)	0.0374 (4)	
H3	0.1760	0.5160	0.6584	0.045*	
C4	0.11908 (12)	0.47672 (8)	0.4495 (2)	0.0373 (4)	
C5	0.09762 (12)	0.48793 (8)	0.2839 (3)	0.0378 (4)	
H5	0.0705	0.4550	0.2182	0.045*	
C6	0.11716 (12)	0.54860 (8)	0.2185 (2)	0.0365 (4)	
N4	0.08177 (14)	0.7500	0.0444 (3)	0.0373 (5)	
H41	0.092 (2)	0.7500	−0.0622 (14)	0.049 (9)*	
H42	0.1081 (14)	0.7162 (8)	0.092 (3)	0.052 (6)*	
N5	−0.15992 (16)	0.7500	0.2772 (3)	0.0368 (5)	
H51	−0.171 (2)	0.7500	0.3843 (14)	0.039 (7)*	
H52	−0.1861 (13)	0.7149 (7)	0.237 (2)	0.041 (5)*	
C7	−0.02674 (18)	0.7500	0.0673 (3)	0.0439 (6)	
H7	−0.0549	0.7881	0.0158	0.053*	
C8	−0.05052 (18)	0.7500	0.2490 (3)	0.0383 (6)	
H8	−0.0219	0.7880	0.3001	0.046*	
O1W	0.2964 (2)	0.7500	0.8852 (3)	0.0739 (7)	
H1W1	0.349 (3)	0.764 (3)	0.845 (7)	0.111*	0.50
H1W2	0.274 (4)	0.721 (2)	0.821 (6)	0.111*	0.50
O2W	−0.0548 (2)	0.2500	0.3099 (4)	0.0840 (8)	
H2W1	−0.007 (3)	0.234 (3)	0.254 (8)	0.126*	0.50
H2W2	−0.031 (4)	0.282 (3)	0.364 (8)	0.126*	0.50

### Atomic displacement parameters (Å^2^)

**Table:**

	*U*^11^	*U*^22^	*U*^33^	*U*^12^	*U*^13^	*U*^23^
O1	0.0535 (8)	0.0364 (7)	0.0551 (9)	−0.0132 (6)	−0.0188 (6)	0.0128 (6)
O2	0.1095 (14)	0.0797 (11)	0.0556 (11)	−0.0613 (11)	−0.0105 (10)	0.0031 (9)
O3	0.1239 (16)	0.0683 (11)	0.0470 (10)	−0.0350 (11)	−0.0241 (10)	0.0044 (9)
O4	0.1063 (13)	0.0433 (9)	0.0739 (13)	−0.0095 (8)	−0.0225 (10)	0.0227 (8)
O5	0.0867 (12)	0.0409 (8)	0.0846 (14)	−0.0227 (8)	−0.0085 (10)	0.0033 (8)
O6	0.0713 (10)	0.0602 (9)	0.0454 (9)	−0.0108 (7)	−0.0001 (7)	−0.0100 (7)
O7	0.0900 (13)	0.0520 (9)	0.0568 (10)	−0.0035 (8)	−0.0298 (8)	0.0076 (8)
N1	0.0470 (8)	0.0382 (8)	0.0453 (10)	−0.0081 (6)	−0.0098 (7)	0.0002 (7)
N2	0.0442 (8)	0.0300 (7)	0.0674 (13)	−0.0006 (6)	−0.0024 (8)	0.0078 (8)
N3	0.0483 (9)	0.0453 (9)	0.0419 (9)	−0.0102 (7)	−0.0065 (7)	0.0009 (8)
C1	0.0308 (7)	0.0314 (8)	0.0430 (10)	−0.0024 (6)	−0.0051 (7)	0.0021 (7)
C2	0.0321 (7)	0.0297 (7)	0.0411 (10)	−0.0021 (6)	−0.0050 (7)	−0.0012 (7)
C3	0.0358 (8)	0.0339 (8)	0.0427 (10)	0.0013 (6)	−0.0045 (7)	0.0054 (7)
C4	0.0343 (8)	0.0274 (7)	0.0501 (11)	−0.0014 (6)	−0.0004 (7)	0.0038 (7)
C5	0.0340 (8)	0.0320 (8)	0.0475 (10)	−0.0034 (6)	−0.0041 (7)	−0.0033 (8)
C6	0.0366 (8)	0.0337 (8)	0.0392 (10)	−0.0020 (6)	−0.0047 (7)	0.0008 (7)
N4	0.0278 (9)	0.0434 (12)	0.0407 (13)	0.000	−0.0018 (9)	0.000
N5	0.0405 (11)	0.0317 (10)	0.0383 (12)	0.000	0.0094 (9)	0.000
C7	0.0315 (11)	0.0627 (17)	0.0377 (14)	0.000	−0.0010 (10)	0.000
C8	0.0399 (12)	0.0387 (12)	0.0363 (14)	0.000	0.0001 (10)	0.000
O1W	0.110 (2)	0.0650 (16)	0.0466 (14)	0.000	−0.0253 (14)	0.000
O2W	0.099 (2)	0.094 (2)	0.0583 (18)	0.000	−0.0144 (15)	0.000

### Geometric parameters (Å, °)

**Table:**

O1—C1	1.251 (2)	C5—C6	1.373 (2)
O2—N1	1.201 (2)	C5—H5	0.9300
O3—N1	1.218 (2)	N4—C7	1.474 (3)
O4—N2	1.227 (3)	N4—H41	0.869 (10)
O5—N2	1.218 (2)	N4—H42	0.864 (9)
O6—N3	1.222 (2)	N5—C8	1.492 (3)
O7—N3	1.223 (2)	N5—H51	0.874 (10)
N1—C2	1.452 (2)	N5—H52	0.863 (9)
N2—C4	1.446 (2)	C7—C8	1.496 (4)
N3—C6	1.454 (2)	C7—H7	0.9599
C1—C2	1.445 (2)	C8—H8	0.9598
C1—C6	1.449 (2)	O1W—H1W1	0.833 (10)
C2—C3	1.373 (2)	O1W—H1W2	0.842 (10)
C3—C4	1.377 (3)	O2W—H2W1	0.847 (10)
C3—H3	0.9300	O2W—H2W2	0.846 (10)
C4—C5	1.382 (3)		
			
O2—N1—O3	120.56 (17)	C6—C5—C4	118.62 (16)
O2—N1—C2	120.41 (17)	C6—C5—H5	120.7
O3—N1—C2	118.99 (15)	C4—C5—H5	120.7
O5—N2—O4	122.85 (18)	C5—C6—C1	124.98 (17)
O5—N2—C4	118.51 (19)	C5—C6—N3	116.01 (16)
O4—N2—C4	118.63 (17)	C1—C6—N3	119.00 (15)
O6—N3—O7	123.42 (18)	C7—N4—H41	107 (2)
O6—N3—C6	117.55 (16)	C7—N4—H42	110.7 (14)
O7—N3—C6	118.99 (17)	H41—N4—H42	111 (2)
O1—C1—C2	124.93 (16)	C8—N5—H51	108.3 (19)
O1—C1—C6	123.89 (17)	C8—N5—H52	110.3 (13)
C2—C1—C6	111.18 (14)	H51—N5—H52	107.7 (17)
C3—C2—C1	124.54 (15)	N4—C7—C8	109.5 (2)
C3—C2—N1	115.98 (16)	N4—C7—H7	109.8
C1—C2—N1	119.48 (14)	C8—C7—H7	109.7
C2—C3—C4	119.21 (17)	N5—C8—C7	111.1 (2)
C2—C3—H3	120.4	N5—C8—H8	109.4
C4—C3—H3	120.4	C7—C8—H8	109.4
C3—C4—C5	121.40 (15)	H1W1—O1W—H1W2	108.3 (18)
C3—C4—N2	119.49 (18)	H2W1—O2W—H2W2	106.5 (17)
C5—C4—N2	119.12 (16)		
			
O1—C1—C2—C3	177.84 (17)	O4—N2—C4—C5	176.88 (18)
C6—C1—C2—C3	−2.6 (2)	C3—C4—C5—C6	−2.0 (3)
O1—C1—C2—N1	−2.2 (3)	N2—C4—C5—C6	178.14 (15)
C6—C1—C2—N1	177.34 (15)	C4—C5—C6—C1	1.6 (3)
O2—N1—C2—C3	158.7 (2)	C4—C5—C6—N3	−179.11 (16)
O3—N1—C2—C3	−19.0 (3)	O1—C1—C6—C5	−179.87 (17)
O2—N1—C2—C1	−21.3 (3)	C2—C1—C6—C5	0.6 (2)
O3—N1—C2—C1	161.01 (18)	O1—C1—C6—N3	0.9 (3)
C1—C2—C3—C4	2.4 (3)	C2—C1—C6—N3	−178.70 (15)
N1—C2—C3—C4	−177.56 (15)	O6—N3—C6—C5	−37.8 (2)
C2—C3—C4—C5	0.1 (3)	O7—N3—C6—C5	140.02 (18)
C2—C3—C4—N2	179.96 (16)	O6—N3—C6—C1	141.56 (16)
O5—N2—C4—C3	175.99 (18)	O7—N3—C6—C1	−40.6 (2)
O4—N2—C4—C3	−3.0 (3)	N4—C7—C8—N5	180.0
O5—N2—C4—C5	−4.1 (3)		

### Hydrogen-bond geometry (Å, °)

**Table:**

*D*—H···*A*	*D*—H	H···*A*	*D*···*A*	*D*—H···*A*
N4—H41···O2W^i^	0.87 (1)	2.06 (2)	2.872 (4)	156 (3)
N4—H42···O1	0.86 (1)	1.98 (1)	2.815 (2)	164 (2)
N4—H42···O7	0.86 (1)	2.41 (2)	2.9166 (16)	118.(2)
N5—H51···O1W^ii^	0.87 (1)	1.91 (1)	2.778 (3)	176 (3)
N5—H52···O1^iii^	0.86 (1)	2.18 (2)	2.892 (2)	140.(2)
N5—H52···O2^iii^	0.86 (1)	2.34 (2)	3.018 (3)	136.(2)
C3—H3···O6^iv^	0.93	2.53	3.337 (3)	145
C7—H7···O2^v^	0.96	2.39	3.128 (3)	134
C7—H7···O7^vi^	0.96	2.56	3.1203 (19)	117
O1W—H1W2···O3	0.84 (1)	2.21 (3)	3.008 (2)	159 (6)
O1W—H1W2···O2	0.84 (1)	2.52 (4)	3.243 (3)	145 (5)
O2W—H2W2···O5	0.85 (1)	2.26 (2)	3.082 (2)	163 (7)

Symmetry codes: (i) −*x*, −*y*+1, −*z*; (ii) *x*−1/2, *y*, −*z*+3/2; (iii) *x*−1/2, *y*, −*z*+1/2; (iv) *x*, *y*, *z*+1; (v) *x*−1/2, −*y*+3/2, −*z*+1/2; (vi) *x*, −*y*+3/2, *z*.

## References

[bb1] Allen, F. H., Kennard, O., Watson, D. G., Brammer, L., Orpen, A. G. & Taylor, R. (1987). *J. Chem. Soc. Perkin Trans. 2*, pp. S1–19.

[bb2] Allen, F. H., Johnson, O., Shields, G. P., Smith, B. R. & Towler, M. (2004). *J. Appl. Cryst.* **37**, 335–338.

[bb3] Harrison, W. T. A., Bindya, S., Ashok, M. A., Yathirajan, H. S. & Narayana, B. (2007). *Acta Cryst.* E**63**, o3143.

[bb4] Macrae, C. F., Edgington, P. R., McCabe, P., Pidcock, E., Shields, G. P., Taylor, R., Towler, M. & van de Streek, J. (2006). *J. Appl. Cryst.* **39**, 453–457.

[bb5] Muthamizhchelvan, C., Saminathan, K., SethuSankar, K., Fraanje, J., Peschar, R. & Sivakumar, K. (2005*a*). *Acta Cryst.* E**61**, o1546–o1548.

[bb6] Muthamizhchelvan, C., Saminathan, K., SethuSankar, K., Fraanje, J., Peschar, R. & Sivakumar, K. (2005*b*). *Acta Cryst.* E**61**, o2887–o2890.

[bb7] Muthamizhchelvan, C., Saminathan, K., SethuSankar, K., Fraanje, J., Peschar, R. & Sivakumar, K. (2005*c*). *Acta Cryst.* E**61**, o2987–o2989.

[bb8] Narayana, B., Sarojini, B. K., Prakash Kamath, K., Yathirajan, H. S. & Bolte, M. (2008). *Acta Cryst.* E**64**, o117–o118. 10.1107/S1600536807062599 PMC2915188 21200681

[bb9] Oxford Diffraction (2007). *CrysAlis PRO* and *CrysAlis RED* Oxford Diffraction Ltd, Abingdon, England.

[bb10] Sheldrick, G. M. (2008). *Acta Cryst.* A**64**, 112–122. 10.1107/S0108767307043930 18156677

[bb11] Spek, A. L. (2009). *Acta Cryst.* D**65**, 148–155. 10.1107/S090744490804362X PMC2631630 19171970

[bb12] Subashini, A., Muthiah, P. T., Bocelli, G. & Cantoni, A. (2006). *Acta Cryst.* E**62**, o3847–o3849.

[bb13] Swamy, M. T., Ashok, M. A., Yathirajan, H. S., Narayana, B. & Bolte, M. (2007). *Acta Cryst.* E**63**, o4919.

